# Detection and manipulation of methylation in blood cancer DNA using terahertz radiation

**DOI:** 10.1038/s41598-019-42855-x

**Published:** 2019-04-23

**Authors:** Hwayeong Cheon, Jin Ho Paik, Moran Choi, Hee-Jin Yang, Joo-Hiuk Son

**Affiliations:** 1 Department of Physics, University of Seoul, Seoul, 02504 Republic of Korea; 2 Department of Pathology, Seoul National University Bundang Hospital, Seong-Nam, 13620 Republic of Korea; 3 Department of Neurosurgery, SMG-SNU Boramae Medical Centre, Seoul, 07061 Republic of Korea

**Keywords:** Cancer therapy, DNA methylation, Biomedical engineering, Terahertz optics, Optical manipulation and tweezers

## Abstract

DNA methylation is a pivotal epigenetic modification of DNA that regulates gene expression. Abnormal regulation of gene expression is closely related to carcinogenesis, which is why the assessment of DNA methylation is a key factor in cancer research. Terahertz radiation may play an important role in active demethylation for cancer therapy because the characteristic frequency of the methylated DNA exists in the terahertz region. Here, we present a novel technique for the detection and manipulation of DNA methylation using terahertz radiation in blood cancer cell lines. We observed the degree of DNA methylation in blood cancer at the characteristic resonance of approximately 1.7 THz using terahertz time-domain spectroscopy. The terahertz results were cross-checked with global DNA methylation quantification using an enzyme-linked immunosorbent assay. We also achieved the demethylation of cancer DNA using high-power terahertz radiation at the 1.7-THz resonance. The demethylation degrees ranged from 10% to 70%, depending on the type of cancer cell line. Our results show the detection of DNA methylation based on the terahertz molecular resonance and the manipulation of global DNA methylation using high-power terahertz radiation. Terahertz radiation may have potential applications as an epigenetic inhibitor in cancer treatment, by virtue of its ability to induce DNA demethylation, similarly to decitabine.

## Introduction

Epigenetic modification is the heritable change without alterations in the DNA sequence. Aberrant epigenetic modification occurs in gene-related diseases because the epigenetic mechanism is closely related to gene expression^1–4^. DNA methylation, an important epigenetic modification, regulates gene expression as a gene silencer. Aberrant DNA methylation can lead to abnormal cell growth (cancer)^5,6^. A well-known example of aberrant DNA methylation is the hypermethylation at CpG islands and the global hypomethylation of repetitive DNA sequences, which is observed in cancer DNA^7,8^. Recent studies have reported that there is a high correlation between carcinogenesis and aberrant DNA methylation in individual genomes and on the global genome-wide scale^5,6,9^. Thus, aberrant DNA methylation is a potential biomarker for cancer, and the manipulation of DNA methylation may be a possible approach to epigenetic therapy^1,10,11^. However, inhibitors of DNA methyltransferases (DNMTs), such as 5-aza-2′-deoxycytidine or 5-azacytidine, which reduce aberrant DNA methylation indirectly in cancer (passive demethylation), have a high risk of side effects because of its poor specificity^12^.

Here, we show that the characteristic frequency for the genome-wide methylated DNA of blood cancer exists in the terahertz (THz) region and that resonant high-power THz radiation can demethylate the genomic DNA directly. THz radiation consists of far-infrared electromagnetic waves ranging from 0.1 to 10 THz (3.3–330 cm^−1^ or 0.4–40 meV). THz radiation has a relatively low photon energy, i.e., not enough to cause ionization, but a high sensitivity for the natural frequencies of chemical bond oscillations of various biomolecules^13^. Because DNA methylation is a chemical modification of DNA without a change in sequence^14^, its spectroscopic characteristics can be sensitively observed in the THz region^15^. The spectroscopic characteristics may also offer insight into the manipulation of DNA methylation. When the methyl-DNA bond is irradiated by THz waves at the natural frequency of the bond oscillations, the energy is preferentially absorbed by the bonding mode, which is regarded as ‘resonance’. The absorption of THz radiation presents resonance peaks in the spectrum, which can be used to identify the methyl-DNA bond and the degree of methylation. Furthermore, high-power THz radiation with sufficient energy can break the methyl-DNA bond by resonant excitation and reduce the degree of methylation.

## The Resonance Frequency of Methylated DNA in Blood Cancer

**Figure 1:**
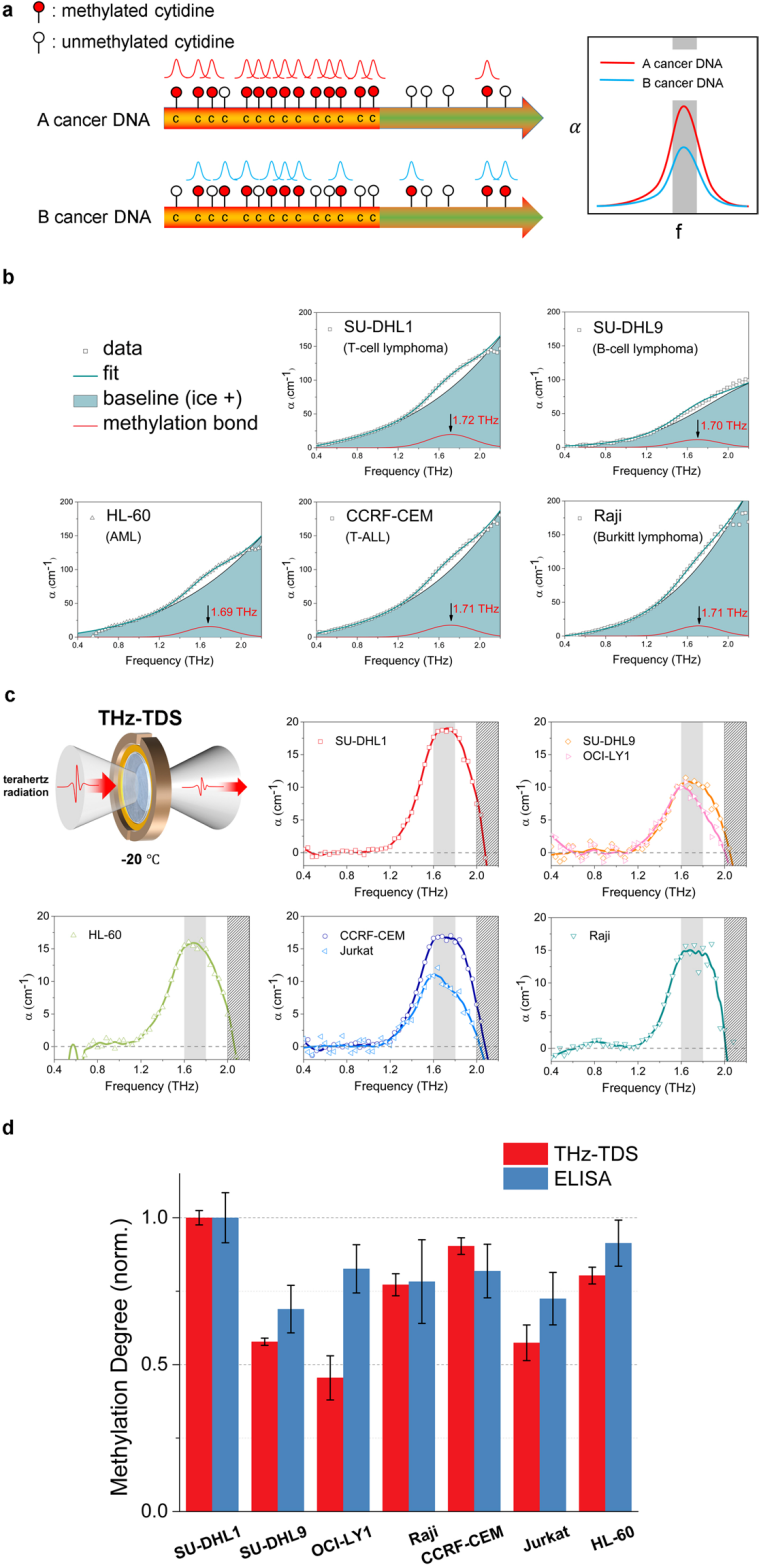
The resonance of the methylated DNA in blood cancer cell lines and the quantification of DNA methylation degree. (**a**) The expected resonance peaks of methylated DNA in the THz spectrum. The THz resonance peaks stem from multiple absorption curves in the same frequency location. The intensity of the THz resonance peak represents the amount of the methyl-DNA bonds in cancer DNA. (**b**) Baseline correction to clarify the resonance peak in five cancer types (in the clockwise direction, T-cell lymphoma, B-cell lymphoma, Burkitt lymphoma, T-ALL, and AML). The measured data were fitted well with the superposition of two Gaussian functions. One of them is the baseline (emerald area) and represents the background materials (e.g., ice, the structure of DNA), and another function is the projected methyl-DNA bond (red line). According to the results, the projected methylation bond in the blood cancer samples shows the resonance at approximately 1.7 THz. (**c**) The resonance peaks of methylated DNA and baseline correction to clarify the peaks in different blood cancer cell lines. In the clockwise direction, the cancers are T-cell lymphoma (SU-DHL1), B-cell lymphoma (SU-DHL9, OCI-LY1), Burkitt lymphoma (Raji), T-ALL (CCRF-CEM, Jurkat), and AML (HL-60). Each datum was obtained by the average of three independent waveforms taking a mean value of three measurements at a point. The centre of the peaks is approximately 1.7 THz, and the average FWHM is 0.5 THz. The amplitudes of the peaks range from 10 to 20 cm^−1^, depending on the type of cancer cell line. (**d**) Verification of the DNA methylation degree measured by THz-TDS (red bar) by comparing the results with those obtained from the global DNA methylation quantification using the ELISA method (blue bar). The ELISA data were obtained by averaging values from six different measurements. Error bars indicate the standard deviation (THz-TDS < 0.075, and ELISA < 0.091). The THz results mostly agree with the ELISA method, which implies that the global DNA methylation degree could be measured directly using the THz-TDS quantification technique.

In order to successfully detect and manipulate DNA methylation, the resonance frequency of the methyl-DNA bond should be found first. We used a THz time-domain spectroscopy (THz-TDS) system to identify the specific resonance frequency in the DNA of blood cancer. In our previous study, the characteristic frequency of methyl CpG in some solid cancer genomic DNA was found to be in the THz region, approximately 1.65 THz, and the quantitative values of global DNA methylation varied with the type of solid cancer cell line^15^. The quantification of the resonance offers information on the degree of global DNA methylation because the resonance peak originates from the superposition of multiple absorption curves at the same characteristic frequency, and the amplitude of resonance indicates how many characteristic bonds exist in the DNA. The intensity might be different for different cancer types because the degree of global DNA methylation varies according to cancer type, as some studies have reported the profile of hypermethylation of CpG islands in several cancer cell lines^16^ (Fig. 1a).

We prepared blood cancer cell lines of five different types: T-cell lymphoma (SU-DHL1), B-cell lymphoma (SU-DHL9 and OCI-LY1), Burkitt lymphoma (Raji), T-cell acute lymphoblastic leukaemia (T-ALL, CCRF-CEM and Jurkat), and acute myeloid leukaemia (AML, HL-60). We measured the absorption coefficient (α) of the samples in aqueous solution using a THz-TDS system. The absorption coefficient spectrum of genomic DNA showed the superposition of a small Gaussian peak and a large Gaussian curve, the latter of which served as the baseline. The small Gaussian peak was projected to be methyl-DNA resonance, and the intensity of the peak represented the degree of DNA methylation. The resonance peaks were extracted from the spectrum by the baseline correction method^17^ (Fig. 1b). The centre of the peaks was located at approximately 1.7 THz, with an average full width at half maximum (FWHM) of 0.50 THz. According to the THz-TDS results, the blood cancer samples had various degrees of global methylation ranging from 10 to 20 cm^−1^ in the absorption coefficient by kind, although the resonance peaks were located at a similar frequency (Fig. 1c). We cross-checked the THz results with a commercial global DNA methylation quantification method using the enzyme-linked immunosorbent assay (ELISA), which is widely used to detect and quantify the methylation of genomic DNA^18,19^. The amplitude of the resonance peak at 1.7 THz was set for a representative value of THz results, and both results were normalized to the highest value (SU-DHL1) due to the difference between the measurement techniques. The THz results showed mostly good agreement with the results obtained using the ELISA method, demonstrating that the THz-TDS technique can directly measure the global DNA methylation degree in blood cancer cell lines (Fig. 1d). In summary, the characteristic energy of methylated DNA in blood cancer cell lines was approximately 1.7 THz, and its global methylation degree varied depending on the type of cancer cell line. The quantification technique of global methylation using THz resonance may be more accurate than the ELISA method, as can be inferred from the smaller error bars.

### Manipulation of methylated DNA using its resonance

**Figure 2:**
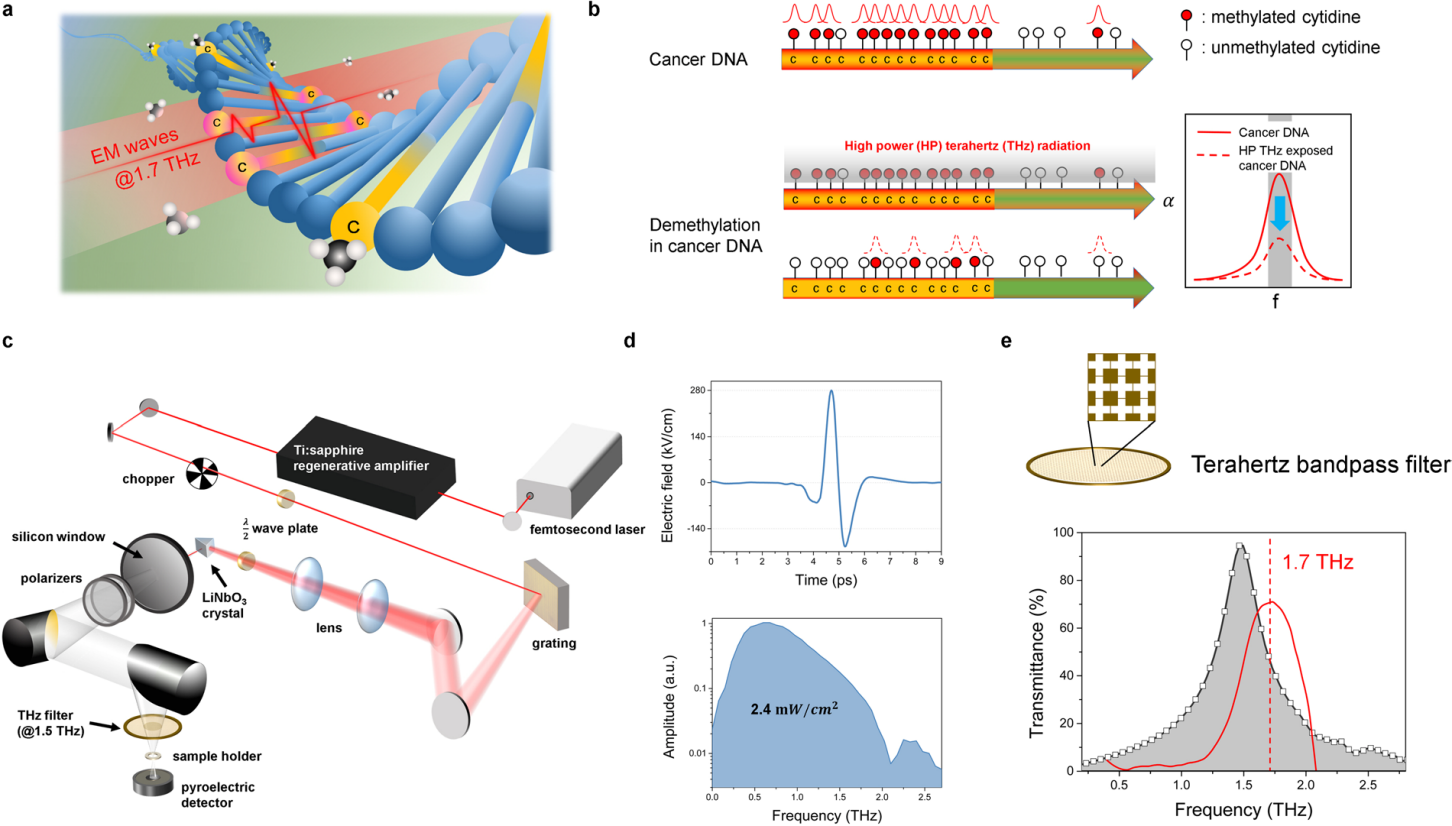
Global demethylation using high-power THz radiation of approximately 1.7 THz and the profiles of high-power THz radiation. (**a**) A schematic illustrating demethylation by THz resonance of the methylated DNA. The 1.7-THz resonant high-power THz radiation has an energy corresponding to that of the methyl-DNA bond. The radiation is expected to influence only the methyl-DNA bond because the THz energy is not sufficient to cause ionization. (**b**) The amplitude decreases in a THz resonance peak by demethylation. The bandwidth-limited THz radiation was applied on a genome-wide scale. If global demethylation is caused by exposure to resonant high-power THz radiation, the amplitude of the resonance peak measured by THz-TDS could decrease after exposure at the resonant frequency. (**c**) Schematic of the high-power THz radiation system used for demethylation. The sample holder in the setup was designed to irradiate THz radiation vertically. (**d**) Temporal and frequency profiles of high-power THz radiation. High-power THz radiation has a pulsed shape in the time domain with a maximum electric field of 280 kV/cm at a 1-kHz repetition rate. The radiation average power density is 2.4 mW/cm^2^ within the valid spectrum, and approximately 8% of the power can pass through a THz bandpass filter. (**e**) A frequency profile of the THz bandpass filter. The centre of resonance peaks for methylated DNA in blood cancer is approximately 1.7 THz (SU-DHL1, red line), but we used a commercial cross-shaped bandpass filter with a 1.5-THz centre frequency (FWHM: 0.42 THz).

The THz technique is useful not only for detecting the methyl-DNA bonds in the genomic DNA but also for manipulating them using high-power radiation. We applied high-power THz radiation at approximately 1.5 THz, which is close to the characteristic frequency of methylated DNA previously determined. The resonance is regarded as a characteristic resonance of the collective vibrations associated with the methyl-DNA bond because the DNA structure and ice, which occupy most of the volume in the sample, do not have a substantial resonance in the THz region^20,21^. If the bandwidth is set at the resonant frequency, and accumulative THz radiation has sufficient energy that is above a critical threshold related to the binding energy of the methylated DNA, the bond between DNA and the methyl group may be broken (Fig. 2a)^22^. We used high-field pulsed THz radiation generated using a 0.6% MgO-doped LiNbO_3_ crystal driven by a 0.8-mJ regenerative amplifier. The average power density of the THz radiation was approximately 2.4 mW/cm^2^, and the THz beam was focused on the centre of the samples (Fig. 2b,c). The spectrum was limited to approximately 1.5 THz (FWHM: 0.42 THz) by a bandpass filter to match the resonance of the methylated DNA. The bandpass filter was a commercially available 1.5-THz cross-shaped filter^23^ and approximately 8% of the power can pass through the filter (Fig. 2d). The bandwidth-limited THz radiation affected the resonance on a global DNA scale. Therefore, if the degree of global methylation is reduced, the intensity of the resonance peaks observed using THz-TDS will be decreased after exposure to high-power THz radiation (Fig. 2e).

### Demethylation of artificially methylated DNA

**Figure 3:**
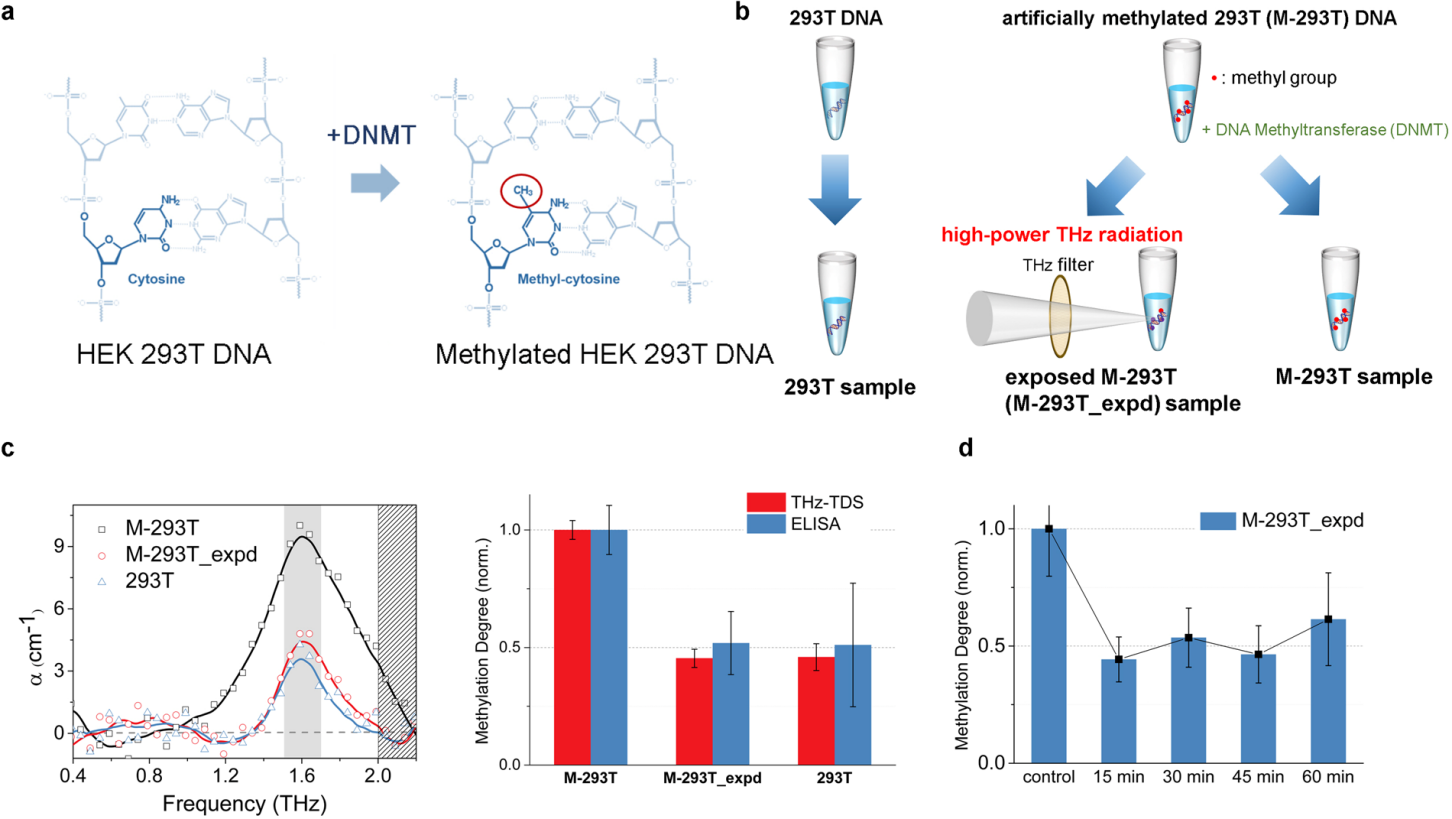
Demethylation of artificially methylated DNA using resonant high-power THz radiation. (**a**) The process of artificial methylation for methylated HEK 293T DNA (M-293T). The extracted genomic DNA from the normal HEK 293 T cell line (293T) was catalysed by DNA methyltransferase (DNMT) into M-293T. (**b**) The samples preparation of the demethylation experiment for artificially methylated 293T DNA. (**c**) The THz resonance spectrum and the degree of DNA methylation of 293T, M-293T, and M-293T_expd which were exposed to resonant high-power THz radiation for 30 minutes. As we predicted based on Fig. 2b, the amplitude of the resonance peak in M-293T DNA was reduced after the exposure. The degree of DNA methylation by THz technique (red bar) was verified using the ELISA method (blue bar). The error bars representing standard deviation are THz-TDS < 0.057 and ELISA < 0.263. (**d**) The efficiency of demethylation vs. the exposed time. The M-293T was divided into five groups which were exposed to high-power THz radiation for the duration of 15, 30, 45, and 60 minutes with one for the control without radiation. The methylation degree was measured by the ELISA method, and the standard deviation was less than 0.202. The efficiency of demethylation was saturated, and there was no substantial change after 15 minutes.

Before the experiment of the blood cancer cell lines, we attempted to reduce the degree of DNA methylation in artificially methylated DNA samples. We prepared genomic DNA samples, which were extracted from human embryonic kidney (HEK) 293T cell lines. We artificially methylated the 293T cell line (M-293T), which was catalysed by DNA methyltransferase (DNMT) (Fig. 3a)^24^. The M-293T DNA sample was irradiated with resonant high-power THz radiation for 30 minutes (M-293T_expd) (Fig. 3b). We measured the degree of methylation in the 293T, M-293T, and M-293T_expd samples using THz-TDS at 1.65 THz and ELISA. The values from the two measurement techniques were compared by normalization. The degree of DNA methylation in the M-293T_expd sample was similar to that in the 293T DNA sample (Fig. 3c). This result indicates that the resonant high-power THz radiation demethylated M-293T DNA and that THz demethylation is a physicochemical reaction induced by a resonance phenomenon. Because some physicochemical reactions are dependent on reaction time, the efficiency of demethylation may depend on the exposure time of THz radiation. To check this relationship, we changed the exposure time of THz radiation by 15-minute increments. As shown in Fig. 3d, the efficiency of demethylation does not depend on the exposure time and the reaction may be complete within 15 minutes with the power level we have used.

### Demethylation of blood cancer DNAs

**Figure 4:**
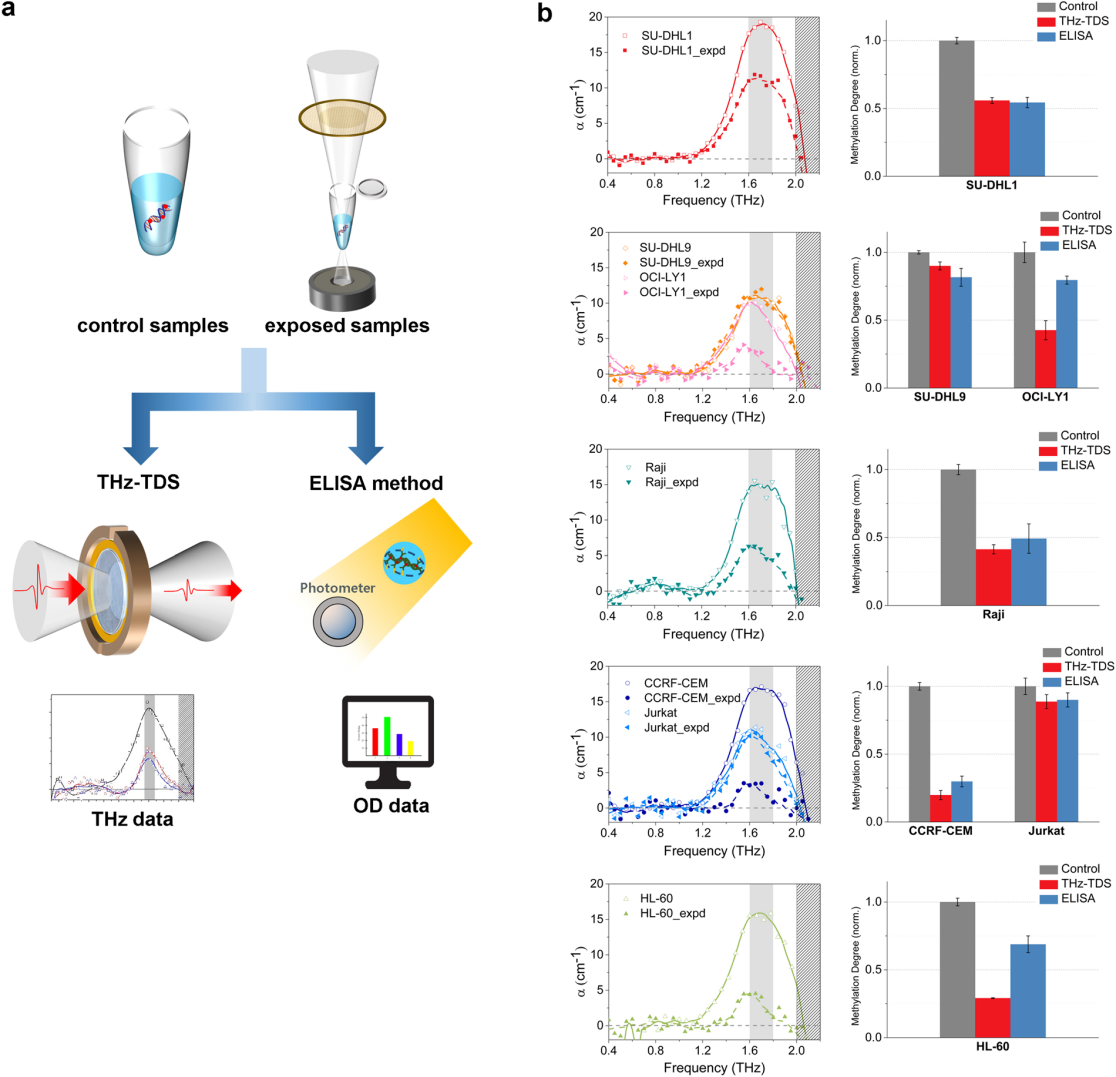
Demethylation of DNA using resonant high-power THz radiation. (**a**) The measurements of the degree of demethylation using the THz-TDS and the ELISA method. (**b**) Demethylation of blood cancer DNA using the resonant high-power THz radiation. The types of blood cancer cell lines were the same as those used in the experiment associated with Fig. 1d. The degree of methylation is mostly in good agreement between the THz (red bar) and ELISA (blue bar) results, which was normalized to compare the results of both techniques. The ratio of reduction ranged from approximately 10% to 70%, depending on the type of cell line. The THz-TDS results were obtained by the average of three independent waveforms taking a mean value of three measurements at a point. The ELISA results were obtained by averaging values from three different measurements. Each measurement was performed twice and averaged. The standard deviations are THz-TDS < 0.071 and ELISA < 0.108.

Because it was demonstrated that high-power THz radiation has the ability to demethylate artificially methylated DNA, we applied this technique to assess the demethylation effect on blood cancer DNA. Using high-power THz radiation, we irradiated the blood cancer DNA samples that had previously undergone DNA methylation degree assessment by THz-TDS. The exposure time was 30 minutes similarly to that in the M-293T experiment, and we measured the degree of methylation using the two previously mentioned techniques (Fig. 4a). The THz quantification was mostly consistent with the ELISA, though there were some differences in OCI-LY1 and HL-60. Although the reduction ratios varied according to cell line (approximately 10–70%), the degree of methylation was substantially decreased in most of the samples (Fig. 4b, Table 1). These results may be a strong evidence that the demethylation of the blood cancer DNA occurs in the specific resonance frequency. For lymphoma cell lines, the degree of demethylation was approximately twofold higher in SU-DHL1 and OCI-LY1 than that in the SU-DHL9. There was a large difference between CCRF-CEM and Jurkat in T-ALL cell lines. The reason for the difference in the reduction ratio was not fully elucidated, but we hypothesize that it is attributed to the difference in collective vibrations according to the arrangement or distribution of DNA methylation. Because we irradiated with a narrow band of THz radiation, having 8% of the total power from THz pulse, in order to affect only the collective vibrations resonantly associated with methyl-DNA bond, it might not have the high energy to disrupt the other structure of DNA^25^. In summary, resonant high-power THz radiation significantly induces global demethylation in genomic blood cancer DNA, and the rate of reduction varies according to cell line.

**Table 1:** Demethylation of the blood cancer DNA in the ELISA results.

	OD^a^ average	Methylation %	Rate of change %
**Control Samples**			
SU-DHL1	1.936^1^, 1.889^1^, 1.721^3^	17.980	
SU-DHL9	1.482^1^, 1.302^2^, 1.365^3^	13.449	
OCI-LY1	1.430^1^, 1.561^2^, 1.475^3^	14.468	
Raji*	1.427^1*^, 1.409^2*^, 1.439^3*^	14.575	
CCRF-CEM*	1.451^1*^, 1.472^2*^, 1.498^3*^	15.097	
Jurkat	1.484^1^, 1.369^2^, 1.445^3^	13.932	
HL-60	1.879^1^, 1.763^2^, 1.695^3^	17.305	
**Exposed Samples**			
SU-DHL1	1.082^1^, 0.978^2^, 1.128^3^	9.777	−45.62
SU-DHL9	1.273^1^, 1.112^2^, 1.179^3^	10.975	−18.40
OCI-LY1	1.207^1^, 1.231^2^, 1.310^3^	11.506	−20.47
Raji*	0.737^1*^, 0.714^2*^, 0.731^3*^	7.206	−50.56
CCRF-CEM*	0.483^1*^, 0.445^2*^, 0.401^3*^	4.186	−72.27
Jurkat	1.384^1^, 1.323^2^, 1.365^3^	12.534	−10.03
HL-60	1.309^1^, 1.284^2^, 1.274^3^	11.907	−31.19
**Control**			
Positive Control	2.487^1^, 2.543^2^, 2.924^3^, 2.518^1*^, 2.399^2*^, 2.173^3*^		
Negative Control	0.006^1^, 0.002^2^, 0.003^3^, 0.070^1*^, 0.035^2*^, 0.031^3^*		
$${\bf{M}}{\bf{e}}{\bf{t}}{\bf{h}}{\bf{y}}{\bf{l}}{\bf{a}}{\bf{t}}{\bf{i}}{\bf{o}}{\bf{n}}\, \% =\,\frac{({\bf{s}}{\bf{a}}{\bf{m}}{\bf{p}}{\bf{l}}{\bf{e}}\,{\bf{O}}{{\bf{D}}}^{{\boldsymbol{a}}}-{\bf{N}}{\bf{e}}{\bf{g}}{\bf{a}}{\bf{t}}{\bf{i}}{\bf{v}}{\bf{e}}\,{\bf{O}}{\bf{D}})/{{\bf{X}}}^{\ast }}{({\bf{p}}{\bf{o}}{\bf{s}}{\bf{i}}{\bf{t}}{\bf{i}}{\bf{v}}{\bf{e}}\,{\bf{c}}{\bf{o}}{\bf{n}}{\bf{t}}{\bf{r}}{\bf{o}}{\bf{l}}\,{\bf{O}}{\bf{D}}-{\bf{N}}{\bf{e}}{\bf{g}}{\bf{a}}{\bf{t}}{\bf{i}}{\bf{v}}{\bf{e}}\,{\bf{c}}{\bf{o}}{\bf{n}}{\bf{t}}{\bf{r}}{\bf{o}}{\bf{l}}\,{\bf{O}}{\bf{D}})\times 10}\times 100$$			
${{\bf{X}}}^{\ast }:{\bf{C}}{{\bf{G}}}^{{\boldsymbol{b}}}\,{\bf{c}}{\bf{o}}{\bf{n}}{\bf{t}}{\bf{e}}{\bf{n}}{\bf{t}}\,({\bf{h}}{\bf{u}}{\bf{m}}{\bf{a}}{\bf{n}}\,{\bf{D}}{\bf{N}}{\bf{A}}:41 \% )$ ^42^			
#^1,2.^: matched controls for each sample			

^a^OD: optical density.

^b^CG: cytosine nucleotide — guanine nucleotide in the linear sequence of bases.

## Discussion

The impact of DNA methylation in molecular cancer therapy has been emphasized in recent years^26,27^. Some researchers have shown that demethylation has a potential in cancer therapeutic applications. DNA methylation inhibitors reduce the risk of cancer progression by preventing the hypermethylation of tumor suppressors or tumor metastasis^28,29^. Several chemical inhibitors and enzymes for DNA demethylation, which remove methyl group indirectly, already have been developed^30–32^. However, there is no current approach using an optical technique for directly manipulating the DNA methylation. Demethylation using THz radiation might have a potential for use in clinical applications as an active demethylation technique; this method is a non-invasive, non-ionizing and non-labelling technique applied to DNA samples. Previous researches have reported that high-power THz radiation could occur the genomic instability^33^, cell division^34^, and chromosome activity^35,36^. Especially, Titova *et al*. showed intense THz pulses alter gene expression in skin, and might potentially induce breaks in DNA and downregulation of gene expression^37,38^. In this study, we suppose that the impact of other DNA structure may be minimal because the filtered THz radiation around the specific resonance frequency has lower power. However, this issue should be checked by additional experiments.

We observed the resonance of the methylated DNA in various blood cancer cell lines at a frequency of 1.7 THz. We further developed this discovery into achieving demethylation by breaking the methyl-DNA bond using the resonant high-power THz radiation. Although we demonstrated the demethylation of cancer DNA from blood cancer cell lines, the molecular dynamics have not been clearly elucidated. Nevertheless, this ‘THz epigenetic scissors’ might serve as a potential cancer therapeutic application similarly to decitabine because THz manipulation of DNA methylation is a controllable active demethylation technique which directly remove the methyl group from DNA.

## Methods

No statistical methods were used to predetermine the sample size. No samples were excluded from the data analysis. Ethical approval for this study was obtained from institutional boards at Seoul National University Boramae Medical Centre and Bondang Hospital. All experiments were approved and performed in accordance with relevant guidelines and regulations by University of Seoul, and Seoul National University Boramae Medical Centre and Bondang Hospital.

### Terahertz time-domain spectroscopy system

The THz time-domain spectroscopy (THz-TDS) system used a Ti:sapphire femtosecond oscillator (Synergy; Spectra-Physics, CA, USA) pumped by a 10-W Verdi diode laser. The oscillator generated a 10-fs pulse laser beam with an 80-MHz repetition rate at a wavelength of 800 nm. To operate the THz-TDS system, the laser beam was separated into two beam lines by a polarizing beam splitter, and each beam was sent toward the emission or detection part of the system. The beam in the emitter was incident at 78° on the surface of a p-InAs crystal for maximum THz intensity, and THz radiation was generated on the crystal using the photo-Dember effect. The THz radiation was collected and transformed into a parallel beam by a parabolic mirror in front of the crystal. The THz beam was focused on the sample holder by a pair of THz lenses (Tsurupica; Microtech Instrument Inc., OR, USA), through which the THz beam is highly transmitted. The THz beam passed through the sample holder and was focused again on the centre of the 5-μm-gap, parallel-line, photoconductive antenna (PCA) detector (PCA-40-06-10-800-h; Batop, Jena, Germany) by another parabolic mirror and a hyperhemispherical silicon lens on the PCA. The focused THz beam combined with the laser beam in the detector, and the laser beam probed the THz signal on the PCA (Supplementary Fig. 1a). The THz-TDS system was operated under 2% humidity during the measurement because THz radiation is highly absorbed by water vapour and the low-temperature sample holder may cause frost on the surface of a window in the sample holder. The sample holder was customized to freeze aqueous solution stably^15^.

The liquid samples were dropped on a z-cut quartz window and covered with an upper window that was composed of hydrophobic material (Teflon) and used to flatten the surface of samples. The quartz window highly transmitted THz radiation (approximately 80%) and had a constant refractive index (~2.1). The loss of THz radiation was minimized because the refractive index is similar to that of ice. The upper window was treated to have a hydrophobic surface with a contact angle larger than 100°, which prevented the liquid sample from sticking to the surface. To freeze the samples, the temperature was maintained at −20 °C (253 K) ± 0.05 °C for 5 minutes using the window that was in contact with a pair of thermoelectric coolers. The sample width was maintained with a 300-${\rm{\mu }}m$ copper spacer during the freezing process. The upper window was removed after the sample was completely frozen. The frozen liquid sample at −20 °C was attached to the quartz window under low humidity, and the same temperature was maintained during the measurements (Supplementary Fig. 1b).

### High-power terahertz exposure system

The high-power THz radiation system consists of an oscillator (Tsunami; Spectra-Physics, CA, USA) that generates a seed beam, a regenerative amplifier (Spitfire; Spectra-Physics) that amplifies the laser beam, and a THz generation system. The Ti:sapphire oscillator generated 35-fs laser pulses at an 800-nm central wavelength with an 80-MHz repetition rate, which was pumped by a 5-W pump laser (millennia Vs; Spectra-Physics) at a central wavelength of 532 nm. The laser beam from the oscillator was amplified to 0.8 mJ of energy per pulse by the Ti:sapphire regenerative amplifier. The repetition rate was also changed to 1-kHz at a central wavelength of 800 nm using a 9-W pump laser (Empower; Spectra-Physics). The pulse front of the amplified laser beam was tilted to 62° by a grating with a groove number of 2000 mm^−1^ and two cylindrical lenses to obtain the maximum power of THz radiation. The tilted-pulse-front beam was incident on a LiNbO_3_ crystal doped with 0.6% MgO, and a high-power THz pulse of 2.4 mW/cm^2^ was collected from the crystal. The residual laser beam was blocked by a wide silicon window, through which THz radiation could penetrate. The high-power THz radiation was gathered by a parabolic mirror and focused vertically to the centre position of the samples by another parabolic mirror. The focused THz beam was passed through a sample holder, and the power of the THz beam was measured using a pyroelectric detector (T-Rad; Gentec-EO, Quebec City, Canada) with a diameter of 5 mm. The bandwidth of THz radiation was limited by a cross-shaped bandpass filter at a 1.5-THz centre frequency with a 0.42-THz FWHM (Thorlabs, NJ, USA). The bandpass filter passed only the THz waves and blocked other infrared waves, including the 800-nm wave that was used to generate the high-power THz radiation. A microcentrifuge tube, which contained the DNA aqueous solution samples, was held vertically by the sample holder (Supplementary Fig. 2). The temporal shape of the THz beam was obtained using an electro-optical sampling technique with a ZnTe (110 direction) crystal with a thickness of 0.5 mm and an auto-balanced detector (ABL-100; Zomega, NY, USA).

### Method to obtain the optical index

We measured a temporal THz pulse using a THz-TDS system. The temporal THz pulse was obtained by finding the mean among three measured data at each sampling point. The temporal THz pulse was obtained by finding the mean among three measured data at each sampling point. We then averaged three independent measurements of the temporal THz pulse to deduce the final data. The temporal data were modified based on the presence of an electric field in the frequency domain by the fast Fourier transform (FFT) algorithm. The absorption coefficient of the samples was calculated by Fresnel’s equation, which compared the reference and sample signals:

1 $${E}_{sam}(\omega )={E}_{ref}(\omega )\,{\exp }(-\frac{d\,\alpha (\omega )}{2}){\exp }(i\frac{2\pi }{\lambda }{n}_{1}(\omega )d)$$

where *E*_*sam*_(*ω*) is the signal that interacts with a sample; *E*_*ref*_(*ω*) is the reference signal; *d* is the thickness of the sample; and α(ω) and *n*_1_(*ω*) are the absorption coefficient and the real part of the complex refractive index of the sample, respectively. From the equation, the absorption coefficient α(ω) can be obtained by:

2 $$\alpha (\omega )=-\frac{2}{d}\,ln({(\frac{{E}_{sam}(\omega )}{{E}_{ref}(\omega )})}^{2})=\frac{4\pi \kappa (\omega )}{{\lambda }_{0}}$$

where *λ*_0_ is the wavelength of THz in a vacuum and *κ*(*ω*) is the imaginary part of the refractive index. A numerical optimization algorithm was applied to solve this relationship using a method proposed by Duvillaret *et al*.^39^.

### Cell culture

Ethical approval for this study was obtained from institutional boards at Seoul National University Boramae Medical Centre and Bondang Hospital. We bought the human embryonic kidney (HEK) 293 T cell line from the central laboratory of Boramae Medical Centre and the seven blood cancer cell lines (SU-DHL1, Jurkat, SU-DHL9, OCI-LY1, CCRF-CEM, Raji, and HL-60) from the Korean Cell Line Bank (Seoul, Republic of Korea) and American Type Culture Collection (ATCC, VA, USA). Cells were cultured in disposable petri dishes with Dulbecco’s modified Eagle’s medium (HyClone, UT, USA) supplemented with 10% foetal bovine serum (HyClone, UT, USA), penicillin-streptomycin 100 U/ml, and kept in an incubator at 37 °C in a humidified atmosphere containing 5% CO_2_. Cells were subcultured once a week by trypsinization (Gibco, Thermo Fisher, MS, USA), followed by gentle re-pipetting and plating at a density of 3 × 10^5^ cells per 100-mm^2^ dish. The culture medium was replaced every 2 days to preserve cell condition.

### Genomic DNA samples

The genomic DNA samples were prepared using the QIAamp DNA Mini Kit (Qiagen, Hilden, Germany) and the FavorPrep Blood/Cultured Cell Genomic DNA Extraction Mini Kit (FAVORGEN, Ping-Tung, Taiwan) to isolate genomic DNA from cells according to the manufacturer’s protocol. The genomic DNAs were dissolved in distilled water after purification to produce an isolated environment and to remove the noise from other residue. To determine the quantity of DNA obtained, we used a NanoDrop ND-1000 UV-Vis spectrophotometer (Wilmington, DE, USA). The concentration of the blood cancer DNA samples was 500 ± 4 µg/ml, and the concentration of 293 T, M-293T, and exposed M-293T DNA samples was 300 ± 7 µg/ml.

### Separation of the DNA methylation signal from the measured data

Before the baseline correction, the absorption coefficients consist of both the DNA methylation signal and the baseline signal originating from ice, extra DNA structures, and residual materials used to produce the DNA samples. We applied the baseline correction algorithm to separate the DNA methylation signal^17,40^. The software packages for numerical computation and visualization, MATLAB 2013 (MathWorks, MS, USA) and Origin Pro 9 (OriginLab Corp., MS, USA), were used to obtain the DNA methylation resonance frequency. We set a Gaussian function as the baseline because ice has the absorption coefficient of a Gaussian form in the range of 0.1–2.0 THz and occupied the largest volume in the DNA samples. The baseline was subtracted from the measurement data. Then, we obtained the resonance peak of DNA methylation.

### Quantification of the DNA methylation signal

To quantify the global DNA methylation, we used the amplitude of the resonance peak in THz-TDS and the Methylamp Global DNA Methylation Quantification Ultra Kit (Epigentek Inc., NY, USA) to verify the THz results^41^. The DNA methylation quantification kit measured the optical density (OD) to obtain the degree of DNA methylation using the enzyme-linked immunosorbent assay (ELISA) method (Supplementary Fig. 3). The ELISA method can be detected as low as 0.2 ng of methylated DNA in 50 ng of input genomic DNA. We divided the sample into three equal portions and measured the OD data two times. The final data was obtained by averaging six data sets. The intensity of the resonance peak was converted to the degree of DNA methylation for comparison with the OD value obtained using the ELISA method. We defined the comparison value as

3 $${\rm{comparison}}\,{\rm{value}}=\frac{{\rm{the}}\,{\rm{intensity}}\,{\rm{of}}\,{\rm{the}}\,{\rm{resonance}}\,{\rm{peak}}}{{\rm{measured}}\,{\rm{data}}\,{\rm{at}}\,{\rm{the}}\,{\rm{resonance}}\,{\rm{frequency}}}$$

The ELISA method yielded the degree of DNA methylation based on the OD value by:

4 $${\rm{methylation}}=\frac{({\rm{sample}}\,{\rm{OD}}-{\rm{negative}}\,{\rm{OD}})/{{\rm{X}}}^{\ast }}{({\rm{positive}}\,{\rm{control}}\,{\rm{OD}}-{\rm{negative}}\,{\rm{control}}\,{\rm{OD}})\times 10}$$

where OD is the optical density and X^*^ is the CG (cytosine – guanine) content (human DNA: 41%)^42^. The OD values and the density of DNA methylation derived from the ELISA method are shown in Table 1. We normalized the degree of DNA methylation obtained from both methods to the highest value as the reference for direct comparison.

### Artificially methylated HEK 293T DNA

The 293 T DNA was divided into two portions, and one of them was artificially methylated (methylated 293 T, M-293T) using the CpG methyltransferase enzyme (New England Biolabs Ltd., MS, USA) according to the manufacturer’s instructions.

### Demethylation of the M-293T DNA samples

The M-293T DNA was divided into two portions, one of which (M-293T_expd) was exposed to the resonant high-power THz radiation for 30 minutes using the high-power THz system. The 293 T, M-293T, and M-293T_expd samples were separated, and the degree of DNA methylation was measured by the THz-TDS and ELISA techniques. To assess the dependence of exposure time, the M-293T DNA sample was divided into five portions and irradiated with high-power THz radiation for different times at 15-minute increments.

### Demethylation of the blood cancer DNA

The DNA samples of blood cancer for the demethylation experiment were prepared to achieve a concentration of 500 ± 4 µg/ml (M-293T DNA was 300 ± 7 µg/ml). The samples were also divided into two portions, one of which served as the control. The other sample was exposed to resonant high-power THz radiation for 30 minutes. The degrees of methylation in the control and experiment samples were measured and compared using the THz-TDS and ELISA methods.

## Supplementary information

Supplementary information
