# Supplementary material for: Detection and manipulation of methylation in blood cancer DNA using terahertz radiation
Source: Sci Rep. 2019 Apr 23;9:6413. doi: 10.1038/s41598-019-42855-x (PMC6478716; doi:10.1038/s41598-019-42855-x)
Supplement: Supplementary file 1 — Supplementary information [file 41598_2019_42855_MOESM1_ESM.pdf]

Detection and manipulation of methylation in blood cancer DNA using terahertz radiation

Hwayeong Cheon[1], Jin Ho Paik[2], Moran Choi[3], Hee-jin Yang[3†], and Joo-Hiuk Son[1†]

[1]Department of Physics, University of Seoul, Seoul 02504, Republic of Korea

[2]Department of Pathology, Seoul National University Bundang Hospital, Seoul 13620, Republic of Korea

[3]Department of Neurosurgery, SMG-SNU Boramae Medical Centre, Seoul 07061, Republic of Korea

[†]To whom correspondence should be addressed: joohiuk@uos.ac.kr, nsyangdr@gmail.com

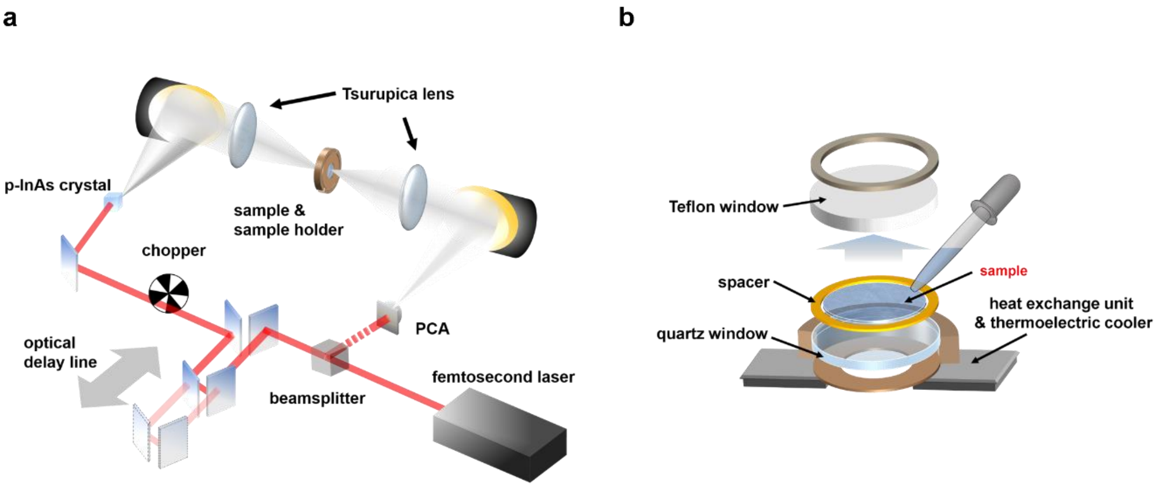

Supplementary Figure 1 THz-TDS setup and the customized sample holder. (a) Schematic of the THz-TDS setup. (b) The detailed structure of the sample holder.

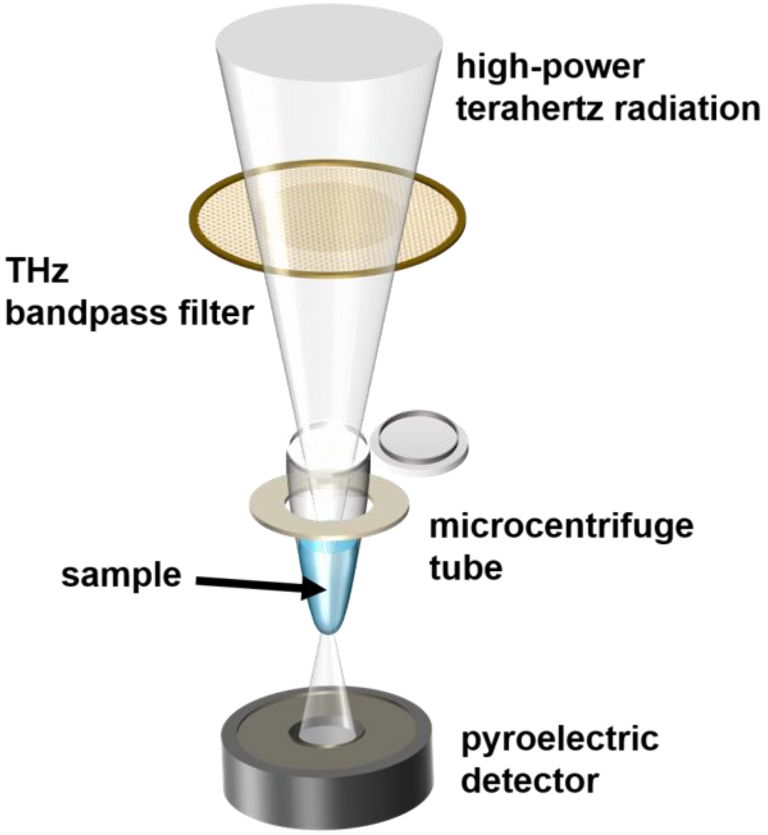

Supplementary Figure 2 The detailed scheme of the sample holder for DNA demethylation, where the bandpass filter limits the bandwidth of terahertz radiation.

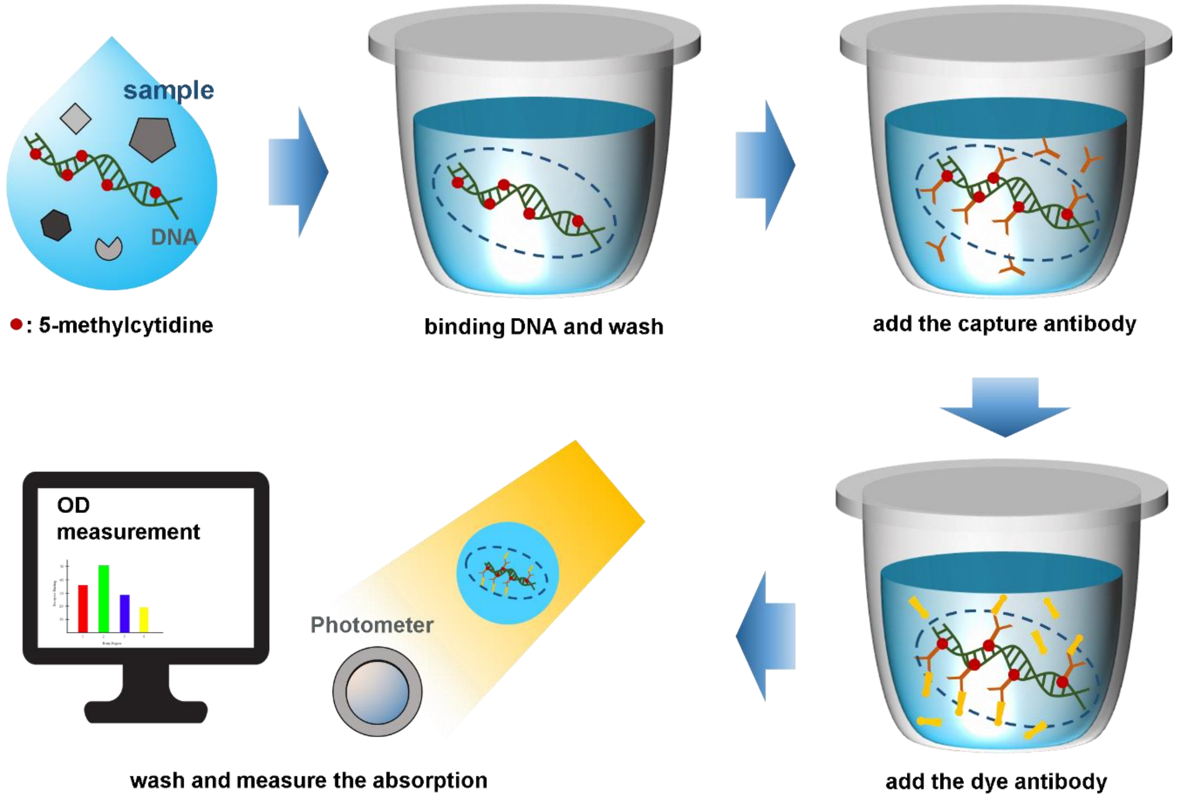

Supplementary Figure 3 The ELISA method using the Methylamp Global DNA Methylation Quantification Ultra Kit.
